# Risk factors for non-puerperal mastitis: a meta-analysis

**DOI:** 10.1186/s12905-025-04110-6

**Published:** 2025-11-18

**Authors:** Hong Liu, Bin Wang, Haobin Wang, Tielin Wang, Jian Wu

**Affiliations:** 1 Department of General Surgery, Center of Breast and Thyroid Surgery, The Third People’s Hospital of Chengdu, Affiliated Hospital of Southwest Jiaotong University, Chengdu, 610031 China; 2 The Second Affiliated Hospital of Chengdu, Chongqing Medical University, Chengdu, 610031 China

**Keywords:** Non-puerperal mastitis, Breast, Risk factors, Meta-analysis

## Abstract

**Background:**

Non-puerperal mastitis (NPM) is a complex inflammatory breast disease with no specific etiology. It is characterized by recurrent episodes and a chronic course. Identifying its risk factors can aid in implementing preventive measures to reduce the incidence of NPM. This meta-analysis aims to determine the risk factors for NPM.

**Methods:**

Relevant studies concerning NPM were retrieved from PubMed, Embase, Cochrane Library, Web of Science, CNKI, and Wanfang databases. Meta-analysis was performed using Revman 5.4 and Stata SE 15.0.

**Results:**

A total of 16 studies with 5402 participants were included, involving 19 risk factors. The following risk factors for NPM were identified: crater nipple, lactation mastitis, breast trauma, breastfeeding time < 6 months, hyperprolactinemia, cardiopathy, hypertension, diabetes, obesity, contraception, smoking, emotional problems, and psychotropic drug use. There was no clear association of NPM with galactostasis, allergic history, alcohol consumption, miscarriage, and divorce or being single.

**Conclusions:**

This study has identified crater nipple, lactation mastitis, breast trauma, breastfeeding duration < 6 months, hyperprolactinemia, cardiopathy, hypertension, diabetes, obesity, contraception, smoking, emotional problems, and psychotropic drug use as risk factors for NPM. For individuals with multiple risk factors, regular breast follow-up is recommended. When breast symptoms occur, early diagnosis and treatment should be implemented based on the associated risk factors to avoid delay in managing the condition.

## Background

Non-puerperal mastitis (NPM) is an inflammatory disease of the breast that occurs outside of lactation. In recent years, the prevalence of NPM has gradually increased. NPM represents 0.3–1.9% of all breast disease cases in the world, while in China, it accounts for 2–5% of all breast lesions [1]. NPM has become one of the benign diseases affecting women’s breast health. NPM is classified into periductal mastitis (PDM)/mammary duct ectasia (MDE) and idiopathic granulomatous mastitis (IGM) [1, 2]. The major manifestations involve breast lumps and abscesses, which frequently recur and lead to persistent breast ulcers and sinus tracts [2]. PDM/MDE is characterized by ductal dilation and plasma cell infiltration, while IGM presents as non-caseating granulomas and microabscesses centered around the lobules of mammary glands [3, 4]. The comprehensive treatment approach for NPM involves identifying the etiology, modifying lifestyle habits, and utilizing a combination of therapies based on lesion location and pathological examination results. These treatment techniques include irrigation of diseased lactiferous ducts and focal debridement, complemented by the timely administration of medications like antibiotics, steroids, and immunosuppressants [5]. Although the exact pathogenesis of NPM remains unclear, smoking, obesity, hyperprolactinemia, and oral contraceptive use have been identified as high-risk factors [6–8]. This study aims to conduct a meta-analysis of the risk factors contributing to NPM by collecting relevant literature from both domestic and international sources. The ultimate objective is to raise awareness of the disease and facilitate its prevention, early diagnosis, and recurrence avoidance.

## Methods

This study has been registered in PROSPERO (CRD42023435142).

### Retrieval strategy

Systematic searches for studies concerning NPM were conducted in PubMed, Embase, The Cochrane Library, Web of Science, CNKI, and Wanfang databases from their inception to April 17, 2023. The search terms consisted of medical mesh terms and keywords related to mastitis and study types. The PubMed search strategy was as follows: (Mastitis [Title/Abstract]) OR (“Mastitis“[Mesh])) AND ((((((Cohort) OR (case-control)) OR (cross-sectional)) OR (randomized controlled trial)) OR (clinical study)) OR (clinical trial))). Additional literature was reviewed through manual searching and reference tracking.

### Inclusion and exclusion criteria

Inclusion criteria: (1) NPM, including PDM, MDE, and IGM; (2) Content: involving risk factors or the association between etiology and NPM. Uniform statistical indicators, such as odds ratio (OR), risk ratio (HR), combined with 95% confidence intervals (CIs) were used to accurately measure the strength of the association between each factor and NPM. At the same time, the definition of significant risk factors should be refined, such as setting relatively high threshold of effect size and strict statistical significance level, so as to improve the clarity and interpretability of the study; (3) Study types: cohort, case-control, cross-sectional, randomized controlled trials, clinical study, clinical trial; (4) English or Chinese publications.

Exclusion criteria: (1) Puerperal mastitis; (2) Mastitis in other species such as cows or mice; (3) Special types of mastitis, e.g., tuberculosis-related mastitis; (4) Studies analyzing only risk factors for NPM without a control group of healthy individuals; (5) Inaccessible full-text articles; (6) Duplicate publications, reviews, comments, or case reports.

### Data extraction and quality assessment

Two independent reviewers screened the articles based on predetermined inclusion and exclusion criteria and extracted data using EndNote X9. The extracted data included first author, year of publication, sample sizes of case and control groups, follow-up duration, risk factors, etiology, and study types. All included studies were case-control studies, and quality assessment was performed using the Newcastle–Ottawa Scale (NOS) [9]. Studies with an NOS score greater than 7 were considered high-quality. In cases of disagreement, consensus was reached through discussion or consultation with a third reviewer.

### Data analysis

Meta-analysis was performed on data using Revman 5.4 and Stata SE 15.0. The odds ratio (OR) from each study was used as an effect measure to calculate the multiple of disease prevalence with risk factors to disease prevalence without risk factors. Heterogeneity was assessed using the Q-test and the statistic I^2^. If I^2^ ≥ 50%, a random-effects model was used; if I^2^ < 50%, a fixed-effects model was employed. Sensitivity analysis was performed by transforming the effect model to assess the stability and reliability of the pooled results. When the number of included studies was ≥ 3, a funnel plot and Egger’s test were used to assess publication bias.

## Results

### Literature search results and quality assessment

**Fig. 1:**
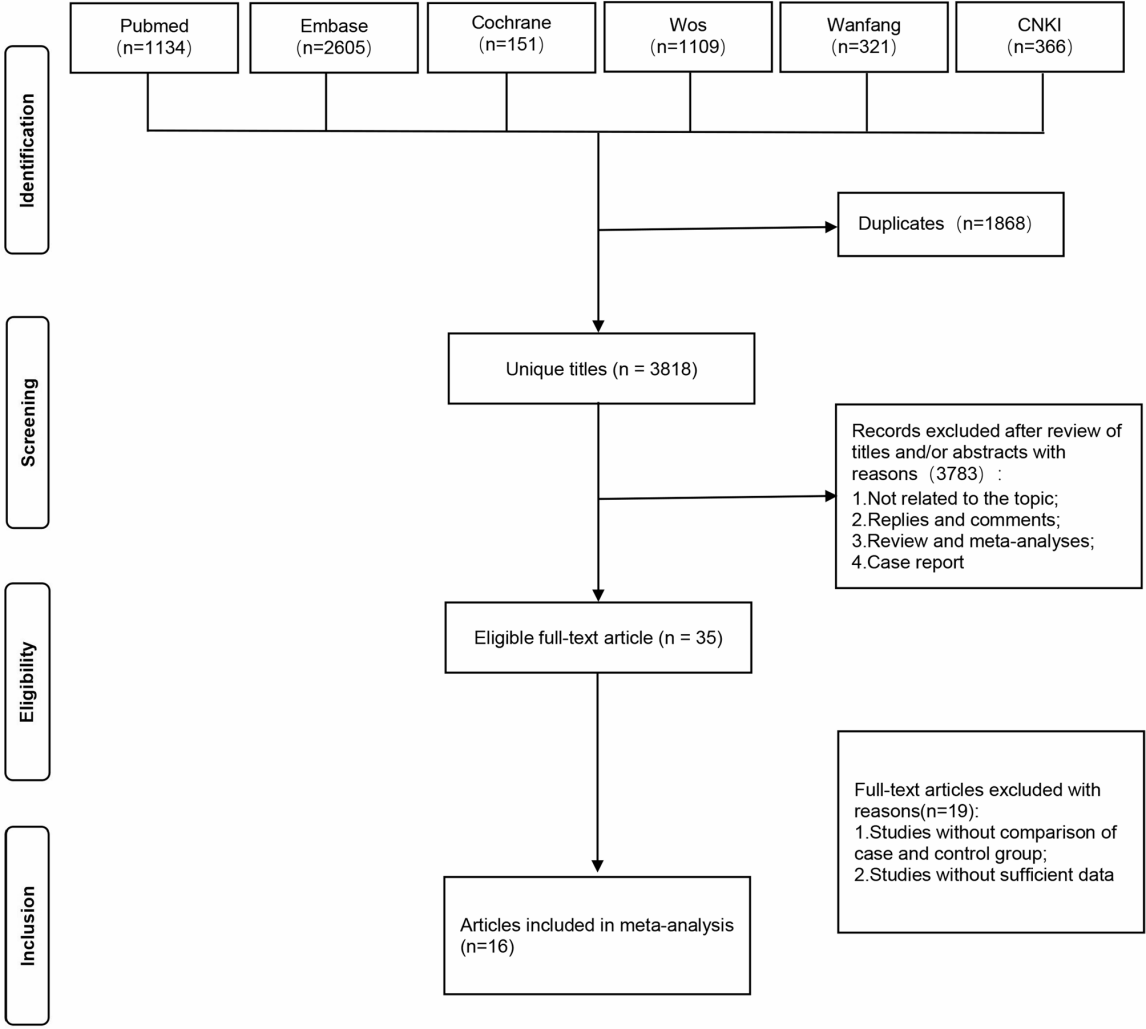
The detailed flowchart for literature screening

This study cited the (PRISMA)2020 [10] reference. A total of 5,686 articles were identified from databases. After removing 1,868 duplicates, 356 records were marked as ineligible by automation tools, and 421 records for other reasons. The full texts of the remaining 44 articles were read according to the inclusion and exclusion criteria. Finally, 16 articles were included [7, 11–25] (12 in Chinese and 4 in English). The literature screening process is summarized in Fig. 1. A total of 1,793 patients with NPM and 3,609 controls were included. The characteristics of the included studies and quality assessment scores are presented in Table 1. The quality of the included studies was assessed by the NOS score scale. The results showed that the NOS scores of the 16 included articles were all greater than 7, indicating high-quality articles.

**Table 1:** Basic characteristics of the included literature

Authors	Study period	Study design	participants (*n*)	Median follow-up (months)	Quality score
			Npm/Control		
Kai Feng 2018 [10]	2011–2016	retrospective	110/110	70	7
Suxiao Jiang 2019 [11]	2016–2018	retrospective	120/220	21	7
Jinling Li 2020 [12]	2014–2019	retrospective	140/140	60	8
Wei Ren 2017 [13]	2012–2016	retrospective	150/150	49	8
Fenli Tian 2019 [14]	2010–2016	retrospective	81/905	72	7
Yanhong Tian 2022 [15]	2021–2022	retrospective	100/100	3	7
Minwen Yan 2020 [16]	2018–2019	retrospective	140/140	11	8
Hailong Yang 2020 [17]	2017–2019	retrospective	50/50	24	8
Weijie Yu 2019 [18]	2017–2018	retrospective	100/100	12	8
Xiang Yue 2023 [19]	2020–2022	retrospective	80/80	32	8
Meng Zao 2021 [20]	2011–2019	retrospective	116/116	96	8
Lu Liu 2017 [21]	2011–2016	retrospective	87/87	51	8
Lu Liu 2016 [7]	2011–2015	retrospective	120/111	26	8
Hanna N 2013[23]	2008–2011	retrospective	85/112	36	8
Haleh Pak 2021[24]	2019–2020	retrospective	30/60	12	8
Liang Shi 2022[25]	2009–2018	retrospective	284/1128	106	8

### Meta-analysis of risk factors for non-puerperal mastitis

The risk factors for NPM can be categorized into breast condition, underlying disease, lifestyle habits, and mental health. The ORs, 95% CIs, and p-values for the risk factors are shown in Table 2.

**Table 2:** Odds ratios for risk factors of non-puerperal mastitis

Factor	Subfactor	OR	95%CI	I^2^	*P*-value	Rerference
Breast condition	Crater nipple	12.6	4.45–35.62	86%	<0.00001	Fenli Tian 2019 [14]; Hailong Yang 2020 [17]; Kai Feng 2018 [10]; Lu Liu 2016 [7]; Meng Zao 2021 [20]; Minwen Yan 2020 [16]; Suxiao Jiang 2019 [11]; Wei Ren 2017 [13]
	Nipple discharge	1.91	0.61–5.98	79%	0.27	Fenli Tian 2019 [14]; Kai Feng 2018 [10]
	Lactation mastitis	2.79	1.67–4.64	72%	<0.0001	Fenli Tian2019 [14];Hailong Yang 2019 [17]Hanna N 2013[23]༛Kai Feng 2018 [10]༛Minwen Yan 2020 [16]༛Suxiao Jiang 2019 [11]
	Breast truma	3.05	1.48–6.40	0	0.003	Meng Zao 2021 [20]; Suxiao Jiang 2019 [11]
	Galactostasis	4.06	0.39–42.71	97%	0.24	Fenli Tian 2019 [14]; Lu Liu 2016 [7]
	Breastfeeding time<6 m	2.13	1.39–3.25	0	0.0005	Fenli Tian 2019 [14]; Hailong Yang 2020 [17]
Underlying disease	Hyperprolactinemia	5.95	2.62–13.50	0	<0.00001	Hailong Yang 2020 [17]; Meng Zao 2021 [20]
	Cardiopathy	17.45	2.26–134.44.26.44	0	0.006	Jinling Li 2020 [12]; Lu Liu 2017 [21]
	Hypertension	19.67	2.58–149.98.58.98	0	0.004	Jinling Li 2020 [12]; Lu Liu 2017 [21]
	Diabetes	17.92	2.32–138.36.32.36	0	0.006	Jinling Li 2020 [12]; Lu Liu 2017 [21]
	Allergic history	1.2	0.65–2.19	0	0.56	Jinling Li 2020 [12]; Lu Liu 2017 [21]
Living habit	Obesity	2.32	1.86–2.89	33%	<0.00001	Hailong Yang 2020 [17]; Kai Feng 2018 [10]; Lu Liu 2016 [7]; Meng Zao 2021 [20]; Weijie Yu 2019 [18]; Wei Ren 2017 [13]; Yanhong Tian 2022 [15]
	Drink	0.89	0.47–1.69	21%	0.73	Kai Feng 2018 [10]; Lu Liu 2016 [7]
	contraception	1.79	1.41–2.28	11%	<0.00001	Fenli Tian 2019 [14]; Hailong Yang 2020 [17]; Haleh Pak 2021[24]; Jinling Li 2020 [12]; Kai Feng 2018 [10]; Lu Liu 2016 [7]; Lu Liu 2017 [21]; Meng Zao 2021 [20]; Suxiao Jiang 2019 [11]; Wei Ren 2017 [13]; Xiang Yue 2023 [19]
	Smoke	2.16	1.71–2.72	0	<0.00001	Fenli Tian 2019 [14]; Hailong Yang 2020 [17]; Hanna N 2013[23]; Kai Feng 2018 [10]; Meng Zao 2021 [20]; Minwen Yan 2020 [16]; Suxiao Jiang 2019 [11]; Xiang Yue 2023 [19]
Mental health	Miscarry	1.34	0.75–2.37	1%	0.32	Hanna N 2013[23];Kai Feng 2018 [10]
	Divorce or Single	1.44	0.91–2.27	49%	0.12	Kai Feng 2018 [10]; Lu Liu 2017 [21]; Minwen Yan 2020 [16]
	Emotional problem	5.42	3.30–8.91	0	<0.00001	Kai Feng 2018 [10]; Meng Zao 2021 [20]; Suxiao Jiang 2019 [11]
	Psychotropic drug	7.76	1.33–45.44	0	0.02	Hailong Yang 2020 [17]; Suxiao Jiang 2019 [11]

### Breast conditions

**Fig. 2:**
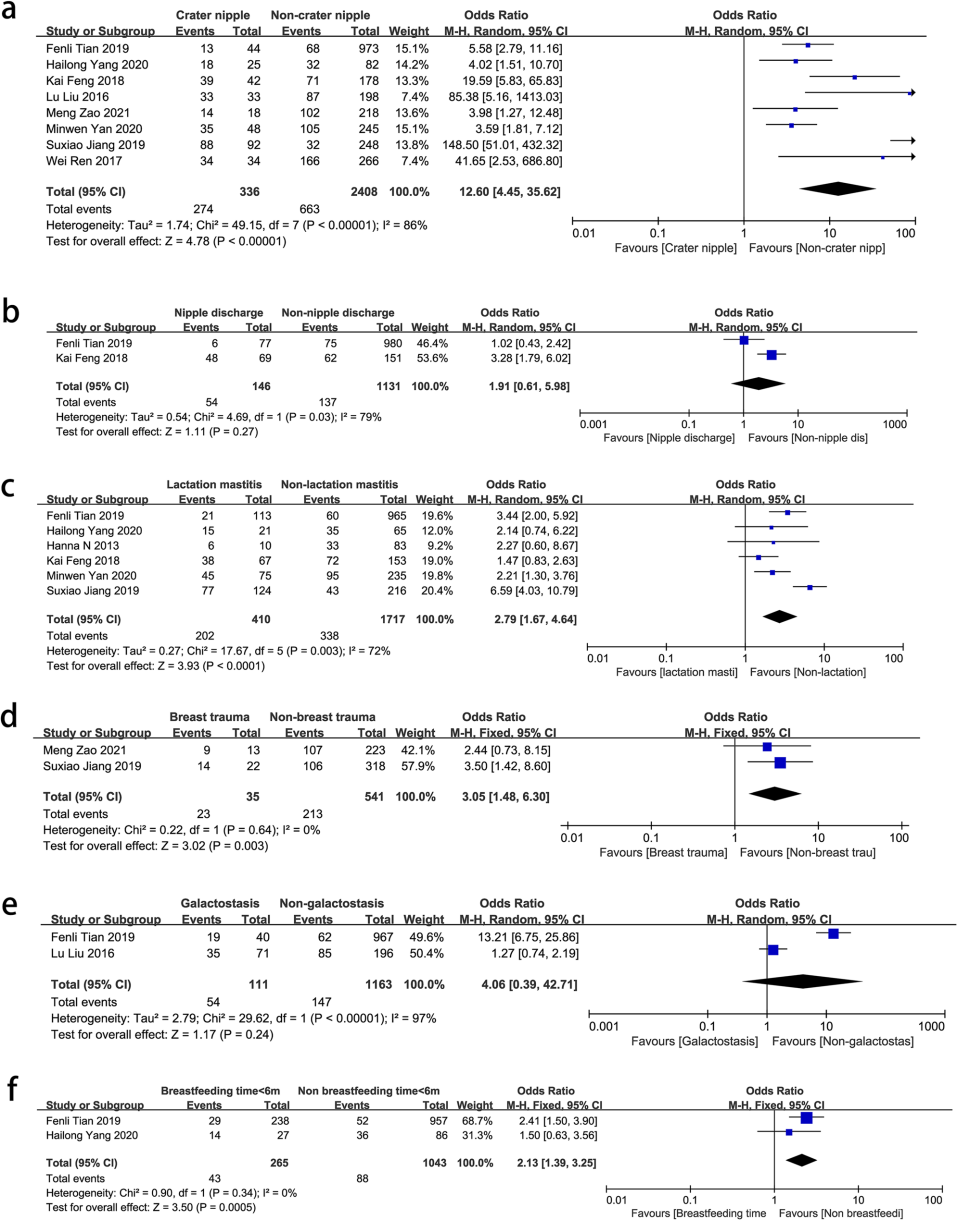
Forest plots of pooled odds ratios (ORs) and 95% confidence intervals (CIs) analyzing risk factors associated with non-puerperal mastitis (breast conditions). **a** Crater nipple vs. non-crater nipple; **b** Nipple discharge vs. no discharge; **c** Lactation mastitis vs. non-lactation mastitis; **d** Breast trauma vs. non-breast trauma; **e** Galactostasis vs. non-galactostasis; **f** Breastfeeding time ≤ 6 months vs. >6 months

Among the breast conditions, crater nipple (OR = 12.6, 95% CI: 4.45–35.62, *P* < 0.00001) was clearly identified as a risk factor for NPM. It could not only affect the breast appearance but also increase the susceptibility to lactation diseases [26] such as lactation mastitis (OR = 2.79, 95% CI: 1.67–4.64, *P* < 0.0001) and breastfeeding time < 6 m (OR = 2.13, 95% CI: 1.39–2.35, *P* = 0.0005), leading to an elevated rate of long-term NPM incidence. Breast trauma (OR = 3.05, 95% CI: 1.48–6.40, *P* = 0.003) had a strong association with an increased incidence of NPM. However, nipple discharge (OR = 1.91, 95% CI: 0.61–5.98, *P* = 0.27) and galactostasis (OR = 4.06, 95% CI: 0.39–42.71, *P* = 0.24) were not correlated with NPM (Fig. 2).

### Underlying diseases

**Fig. 3:**
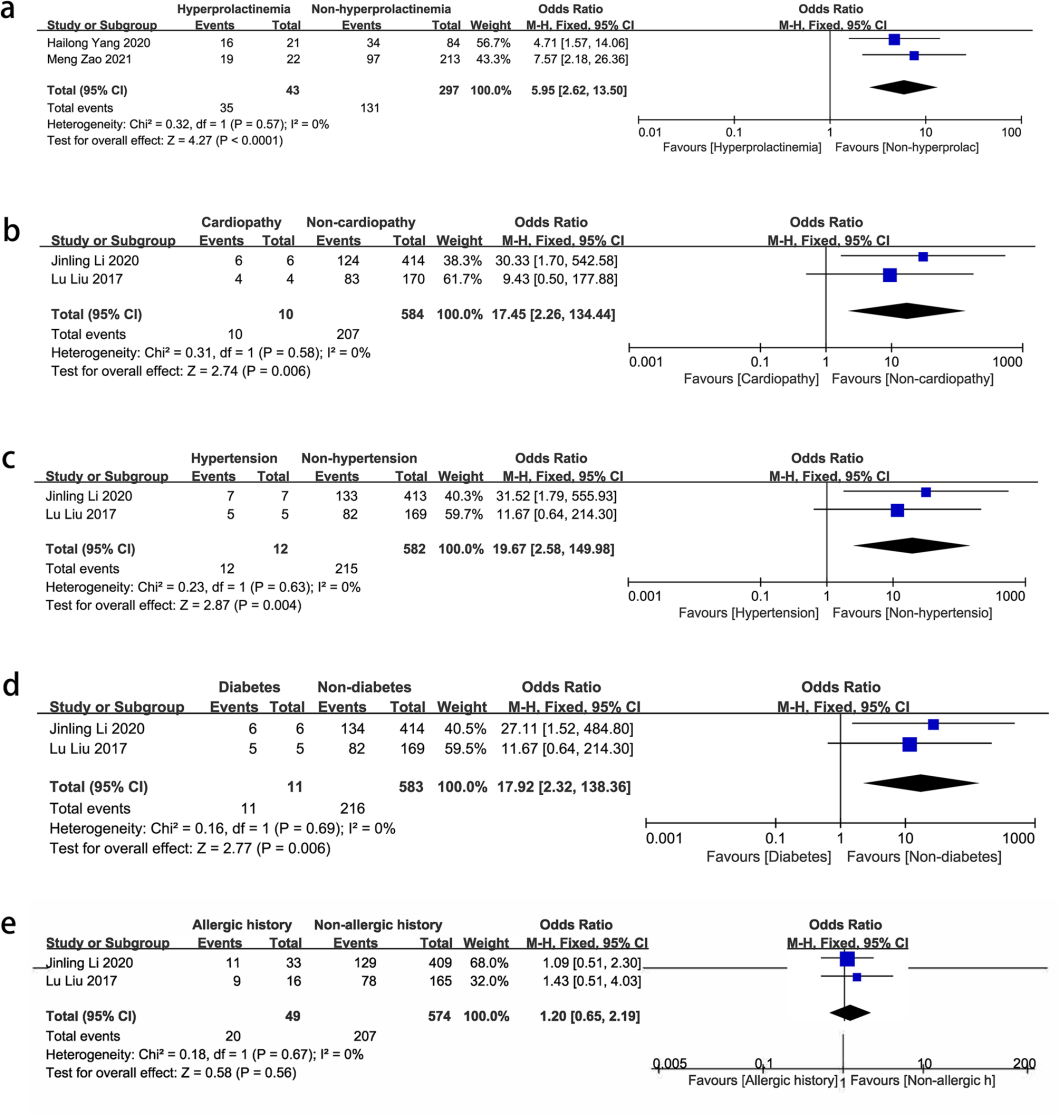
Forest plots of pooled odds ratios (ORs) and 95% confidence intervals (CIs) analyzing risk factors associated with non-puerperal mastitis (underlying diseases). **a** Hyperprolactinemia vs. non- hyperprolactinemia; **b** Cardiopathy vs. non-cardiopathy; **c** Hypertension vs. non-hypertension; **d** Diabetes vs. non-diabetes; **e** Allergic history vs. non-allergic history

Hyperprolactinemia had a significant association with NPM (OR = 5.95, 95% CI: 2.62–13.5, *P* < 0.00001). Cardiopathy (OR = 17.45, 95% CI: 2.26–134.44.26.44, *P* = 0.006), hypertension (OR = 19.67, 95% CI: 2.58–149.98.58.98, *P* = 0.004), and diabetes (OR = 17.92, 95% CI: 2.32–138.36.32.36, *P* = 0.006) also showed correlations with NPM, although they were not established risk factors in previous reports. There was no correlation between allergic history (OR = 1.2, 95% CI: 0.65–2.19, *P* = 0.56) and NPM. (Fig. 3)

### Lifestyle habits

**Fig. 4:**
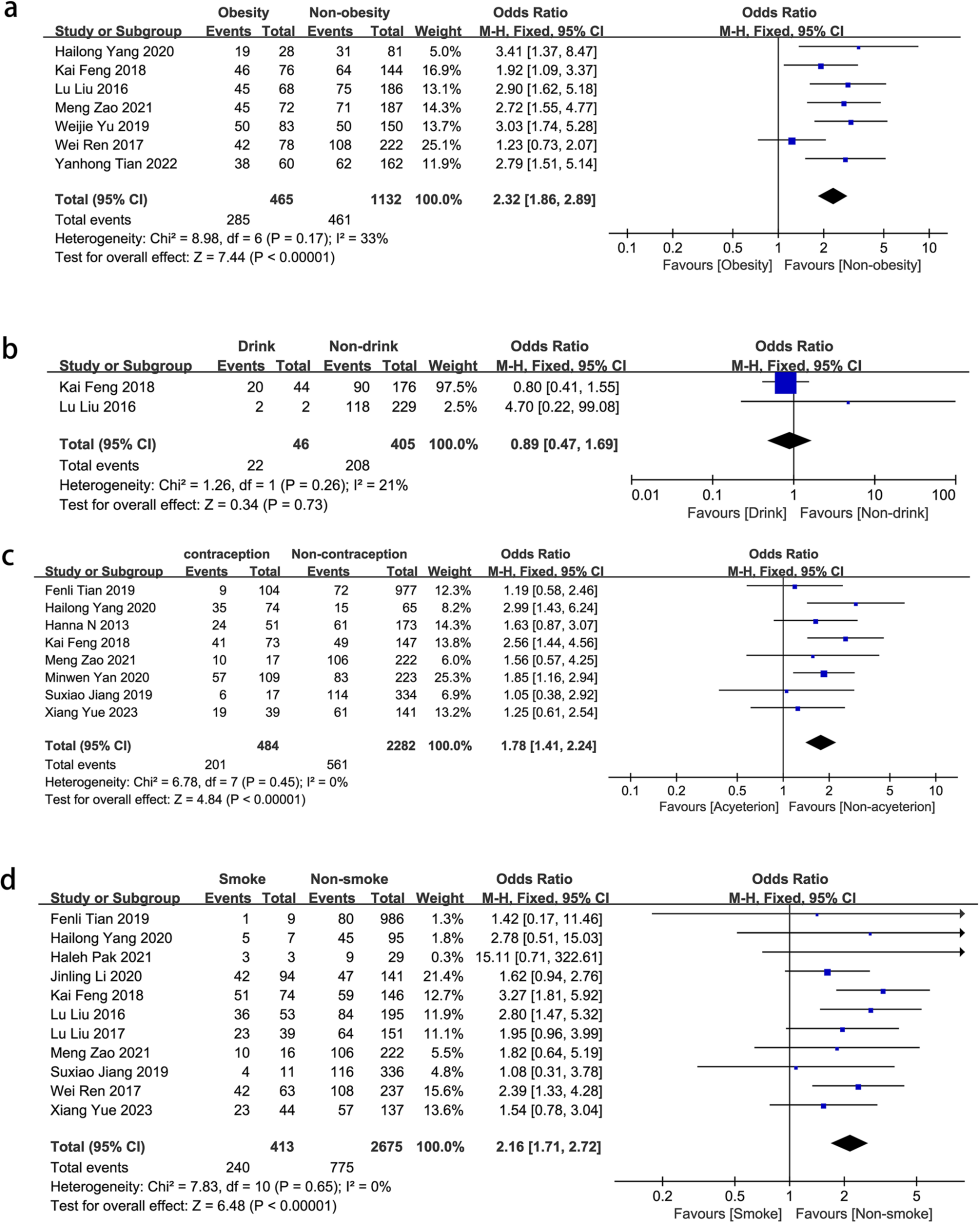
Forest plots of pooled odds ratios (ORs) and 95% confidence intervals (CIs) analyzing risk factors associated with non-puerperal mastitis (lifestyle habits). **a** Obesity vs. non-obesity; **b** Drink vs. non-drink; **c** Contraception vs. non-contraception; **d** Smoking vs. non-smoking

Lifestyle habits are important factors for NPM. Obesity (OR = 2.32, 95% CI: 1.86–2.89, *P* < 0.00001), smoking (OR = 2.16, 95% CI: 1.71–2.72, *P* < 0.00001), and contraception (OR = 1.79, 95% CI: 1.41–2.28, *P* < 0.00001) were associated with a significantly higher prevalence of NPM compared to the normal population. Drinking (OR = 0.89, 95% CI: 0.47–1.69, *P* = 0.73) did not have a significant impact on the prevalence of NPM (Fig. 4).

### Mental health

**Fig. 5:**
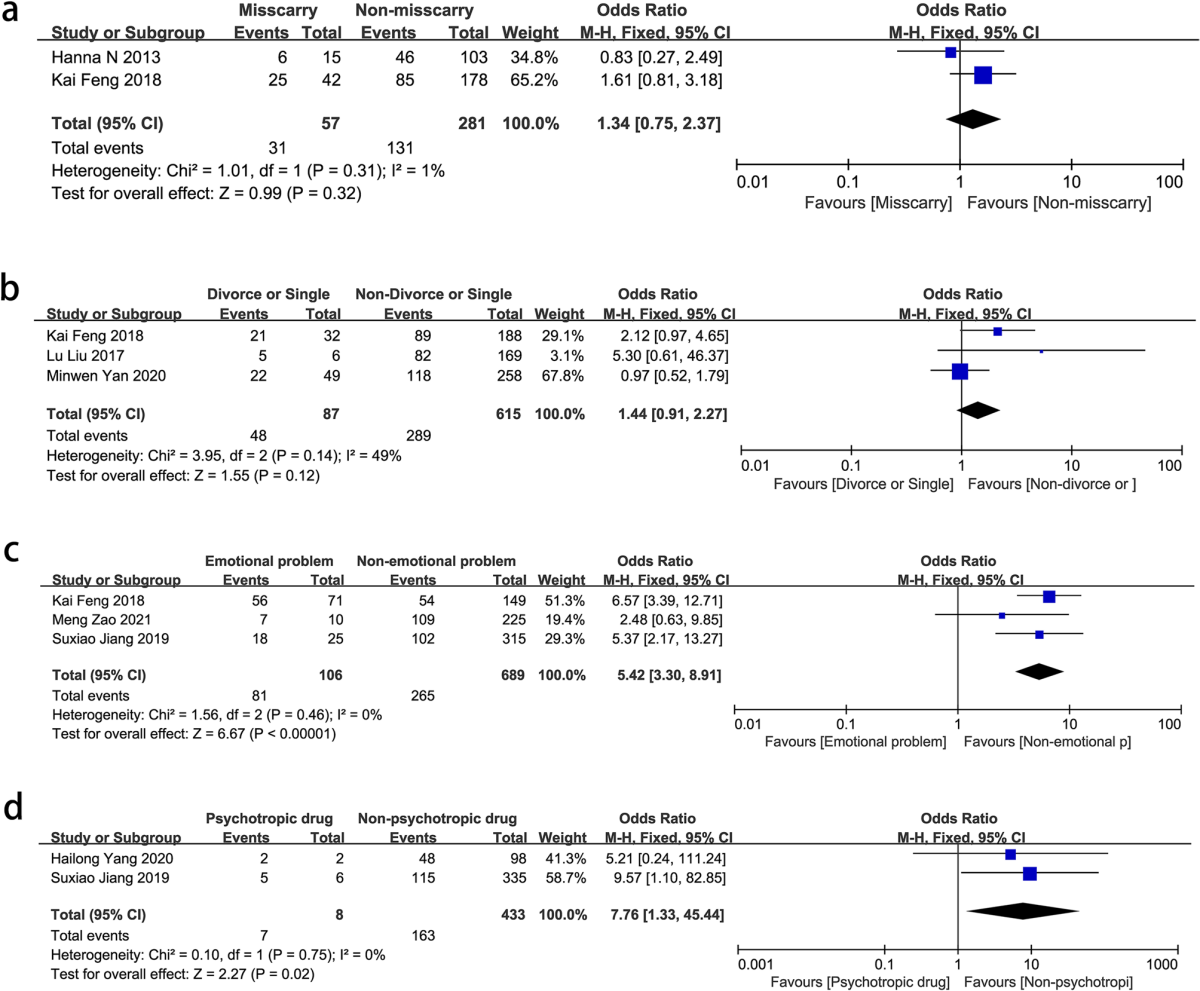
Forest plots of pooled odds ratios (ORs) and 95% confidence intervals (CIs) analyzing risk factors associated with non-puerperal mastitis (mental health). **a** Miscarry vs. non-miscarry; **b** Divorce vs. non-divorce; **c** Emotional problem vs. non-emotional problem; **d** Psychotropic vs. non-psychotropic

Emotional problems (OR = 5.42, 95% CI: 3.30–8.91, *P* < 0.00001) and psychotropic drug use (OR = 7.76, 95% CI: 1.33–45.44, *P* = 0.02) were strongly associated with NPM. Miscarriage (OR = 1.34, 95% CI = 0.75–2.37, *P* = 0.32) and divorce or being single (OR = 1.44, 95% CI: 0.91–2.27, *P* = 0.12) were not correlated with NPM (Fig. 5).

### Sensitivity analysis and publication bias

**Fig. 6:**
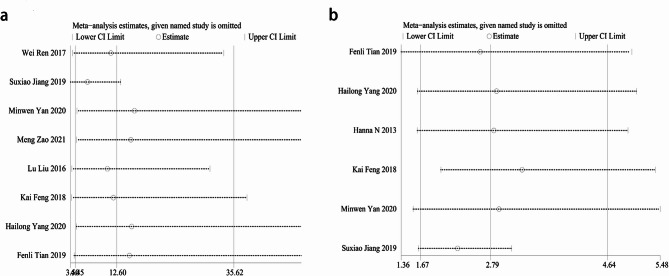
Sensitivity analysis of risk factors associated with non-puerperal mastitis. **a** crater nipple; **b** lactation mastitis

**Fig. 7:**
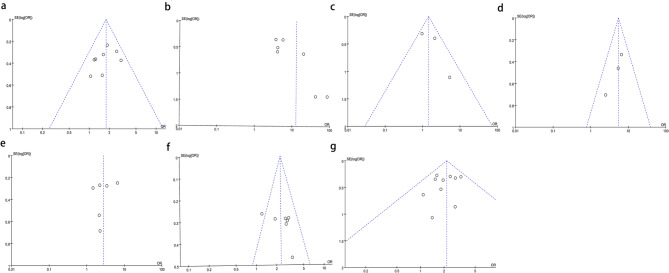
Funnel plot of risk factors associated with non-puerperal mastitis. **a** contraception; **b** crater nipple; **c** divorce or single; **d** emotional problem; **e** lactation mastitis; **f** obesity; **g** smoking

**Fig. 8:**
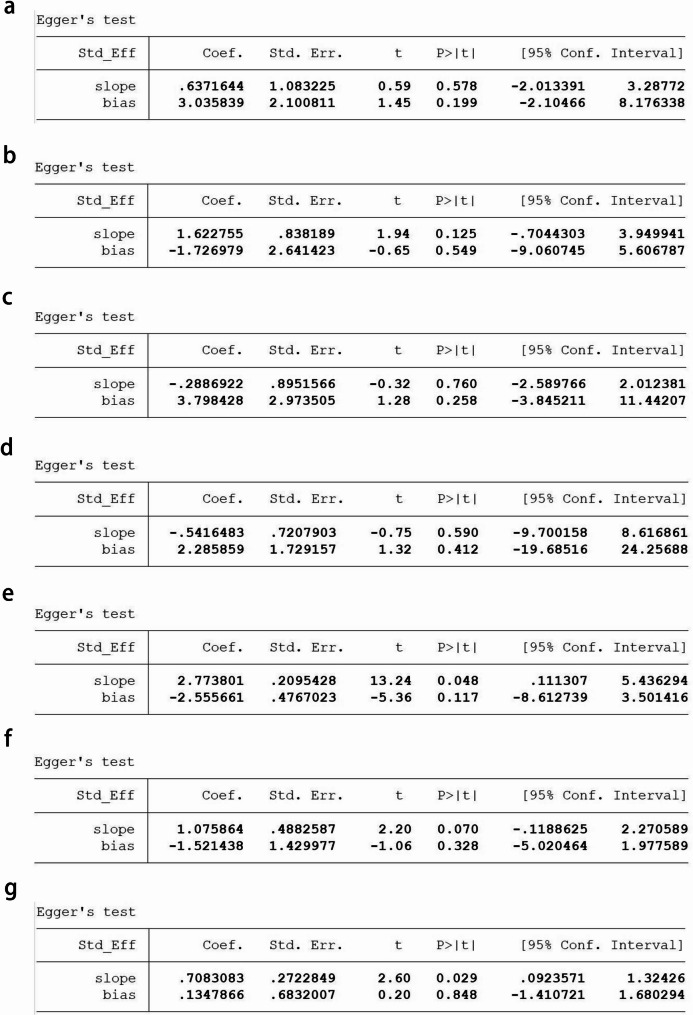
Egger’s test of risk factors associated with non-puerperal mastitis. (**a**) crater nipple, (**b**) lactation mastitis, (**c**) obesity, (**d**) divorce or being single, (**e**) emotional problem, (**f**) contraception, (**g**) smoking

A sensitivity analysis was conducted on the included studies, and no study significantly affected the meta-analysis results, indicating relatively stable meta-analysis results and good overall consistency (Fig. 6). According to the funnel plot (Fig. 7) and Egger’s test (Fig. 8), there was no apparent publication bias. The results of Egger’s test were as follows: crater nipple (*P* = 0.199), lactation mastitis (*P* = 0.549), obesity (*P* = 0.258), divorce or single (*P* = 0.412), emotional problem (*P* = 0.117), contraception (*P* = 0.328), smoking (*P* = 0.848).

## Discussion

Breast condition is an important risk factor for NPM. Women of childbearing age with a history of breastfeeding are susceptible to NPM [27]. During lactation, simple galactostasis does not increase the long-term risk of NPM. However, breast redness, pain, or abscess formation can occur if galactostasis leads to lactation mastitis, often resulting in a shorter duration of breastfeeding (less than 6 months) [28]. Similar to breast trauma, it may be associated with chronic nonspecific lobulitis caused by secretion leakage, granuloma formation, or central purulent necrosis of the lobules [29]. Due to nipple retraction, the opening of the nipple is submerged, and secretions accumulate within the ducts, leading to ductal dilation, ductal inflammation, and recurrent infections [30, 31]. Nipple discharge is often a clinical manifestation of NPM during the period of ductal dilation. It is characterized by multiple duct involvement and discharge of different colors [32] and is not a causative factor for NPM.

The correlation between prolactin and NPM has been confirmed [33]. Yuan [34] conducted a statistical analysis on 64 patients with granulomatous mastitis (GLM) and found that 71.9% of the cases had abnormal serum prolactin levels, which were significantly higher than those in the healthy population. Hyperprolactinemia promotes the secretion of lipids and proteins in the mammary ducts, causing the accumulation of lipid-containing fluid within the ducts and subsequent ductal injury and aseptic inflammation around and within the ducts. Additionally, upon antigen exposure, it also activates an autoimmune response [33, 35, 36]. In cases where pituitary tumors and IGM occur together, surgical removal of the pituitary tumor results in a return to normal serum prolactin levels and complete resolution of inflammatory changes in the breast [37]. It is important to monitor serum prolactin levels before and after the treatment of mastitis to prevent relapse. Antipsychotic medications increase serum prolactin levels by causing pituitary release of prolactin through dopamine receptor blocking or exerting weak dopamine receptor antagonism, further affecting the breast [38]. Patients who are concurrently taking antipsychotic medications and have nipple retraction are more prone to developing NPM [39]. It is crucial to monitor prolactin levels and keep them within the normal range in patients taking antipsychotic medications [40].

Diabetes is an important risk factor for NPM [41]. Among 98 NPM patients, 75% had diabetes at the onset of NPM or developed diabetes within a 5-year follow-up period. The resolution time for abscesses in NPM patients with diabetes was significantly longer compared with those without diabetes [42]. In premenopausal patients with type 1 diabetes and microangiopathy, it is important to differentiate between diabetic mastopathy and breast masses to avoid unnecessary surgeries and treatments [43]. It is recommended to assess diabetes in NPM patients with breast abscess, regardless of family history [44]. This study indicates that hypertension and heart disease are also risk factors for NPM, while an allergic history is not a risk factor. However, due to the limited number of included patients, further research is needed for confirmation. Lifestyle habits have a significant impact on NPM, with smoking and obesity being independent risk factors. Smoking causes periductal inflammation in the breast, which may be associated with the toxic effects of smoking on the ducts, leading to anaerobic bacterial infections around the ducts [45, 46]. In IGM, patients in the active phase have a higher smoking rate compared with those in the stable phase of inflammation (78.9% vs. 38.5%) and the healthy population [47]. The longer the duration of smoking, the greater the likelihood of ductal dilation and ductal inflammation. However, the effects diminish after one year of smoking cessation [32]. Smoking plays a decisive role in recurrent mastitis, and encouraging patients to quit smoking will promote wound healing and reduce relapse [48]. Based on breast color Doppler ultrasonography classification of 100 cases of NPM, when BMI ≥ 24 kg/m^2^, the breasts exhibited a decreased amount of glandular tissues and a higher level of adipose tissues [16]. Furthermore, inflammation increased within the adipose tissues [49], leading to further activation of the immune response [50] and the subsequent development of NPM. Every 10 g/day increase in alcohol intake increases the risk of developing breast cancer by 4%, while the relationship between alcohol consumption and NPM has not been reported. Following oral contraceptive use, the breast tissue became more secretory and the content of the ducts came into contact with the surrounding tissue, leading to a chronic granulomatous reaction [51]. Obesity induces increased biosynthesis of adipose tissue, resulting in elevated estrogen levels similar to those caused by exogenous estrogen intake. It may be a triggering factor for NPM [52].

Some research institutes collect a large amount of data on female patients with NPM through large-scale epidemiological surveys, and use professional psychological assessment scales to comprehensively evaluate their mental health. This study aims to explore whether there is a potential association between the two, such as whether certain psychological states increase the risk of NPM, or vice versa, whether the disease has a negative impact on the mental health of patients [8, 9]. In terms of mechanism exploration, some studies may focus on the deep mechanism of interaction between physiology and psychology. They may try to explain how mental health factors affect the physiological function of breast tissue and then lead to the occurrence and development of NPM using advanced biotechnology, such as gene testing, hormone level determination, and neuroimaging technology. Some other studies ascertain how NPM-related symptoms, such as physical discomfort and pain, feed back into the brain, causing psychological changes [15]. The articles included in this study took into account the impact of mental health problems on NPM. On the one hand, the included studies may have some limitations in sample selection. Most studies tend to focus on specific regions or specific populations, with insufficient representativeness and universality, which makes it difficult to generalize the results to a wider population. On the other hand, the existing literature on intervention measures is relatively weak. Although some studies have confirmed that mental health interventions are helpful to improve the prognosis of NPM patients, the specific intervention programs are not perfect and standardized. The number of abortions had no impact on the development of NPM, but multiple pregnancies reduced the risk of this disease [11]. In the pathogenesis of NPM, emotional issues as a potential risk factor have been increasingly studied, but their specific pathway still needs to be further analyzed. In women with chronic anxiety, depression, or high stress, the neuroendocrine system is unbalanced, where constant sympathetic stimulation leads to increased secretion of catecholamine, which in turn leads to abnormal nipple vasoconstriction. Meanwhile, elevated cortisol levels may suppress immune function and reduce the ability of local tissues to defend against pathogens. This physiological change not only directly weakens the self-repair mechanism of the breast, but also has synergistic effects with traditional risk factors such as mechanical stimulation and hormone fluctuations [11, 12]. For example, when emotional stress is superimposed on the estrogen peak before the menstrual cycle, breast ductal epithelial cells are more susceptible to secondary infection due to microdamage [12]. Although divorce or being single is not a risk factor for NPM, marital status is an important association with depression [53], which is more pronounced in divorced women [54]. In patients with NPM, breasts can deform due to long-term chronic inflammation, resulting in asymmetry and affecting marital relationships. When women suffer from chronic inflammation for a long time and their breast morphology changes, this significant physiological change can easily impact their relationship. The original harmonious and intimate relationship atmosphere may change, and one of them may have complex psychological reactions due to visual intuition, such as anxiety, deep worry and even subconscious resistance. For divorced women, their already fragile emotional connection is more vulnerable to such factors. They may have taken the new relationship as a way to heal the past trauma, but the deformed breast is like an insurmountable barrier to rebuilding trust and deepening intimacy. From the perspective of psychology, breast, as a core secondary sexual characteristic in females, has the symbolic significance of sexy characteristics and charm. Their morphological imbalance will significantly reduce the level of self-awareness in women, resulting in their loss of self-confidence in sexual communication, as well as withdrawal and avoidance behavior. If the partner fails to provide sufficient understanding and support, the contradiction between the two sides will gradually intensify, the frequency of daily communication will be reduced, and the degree of emotional alienation will be deepened. Over time, the strong passion of love in the past may fade away quietly, and the marriage relationship will become nominal [53, 54]. The mental health monitoring of 622 perinatal women showed that a poor psychological state was associated with lactational mastitis [55], which may also increase the risk of future NPM.

This study is the first analysis of risk factors for NPM, identifying high-risk factors with stable results and minimal heterogeneity, without publication bias. However, there are some limitations. First, the majority of studies included in this analysis were conducted in China. This phenomenon may introduce a certain degree of regional bias, which may have an impact on the accuracy and generalizability of the results. In addition, the number of patients included in some studies is too small to cover all kinds of situations, which may lead to some bias in the conclusions. Additionally, all the included studies were retrospective case-control studies, lacking prospective study designs. There are limited studies included in the meta-analysis of some risk factors, with only two studies available. The evidence for diabetes, heart disease, and hypertension as risk factors is still insufficient. Future high-quality prospective studies are needed to provide a more comprehensive and scientific assessment.

During the literature search of this study, a large number of excluded studies seriously increased the workload. More importantly, these excluded studies may hide some valuable information or unique perspectives, but they are not effectively captured due to the limitations of current search strategies. Therefore, we will further optimize the retrieval strategy in future research. First of all, it is necessary to accurately define keywords and expand synonyms. On the one hand, future research should disassemble the research theme in depth and clarify the core concepts and their related forms of expression. On the other hand, it is important to use the thesaurus function of professional databases: Many academic databases provide special controlled vocabulary lists or subject thesauri. Researchers can use these resources to find standardized terms that are closely related to the research topic and incorporate them into the search strategy. Secondly, the Boolean logical operators are flexibly used to combine the search expressions. It is imperative to carefully design the complex search formula containing Boolean logical operators such as “AND”, “OR” and “NOT” according to the research needs. Furthermore, the method of gradual refinement is used to construct the retrieval strategy. Starting from a broader theme, a certain number of basic documents are obtained as samples. After analyzing the characteristics of these documents, more specific restrictions are added to gradually narrow the search scope to the ideal level. Finally, cross-database retrieval and multi-source validation are needed. The advantages of different types of databases should be integrated. After the same retrieval strategy is used among different databases, the differences in the obtained results should be compared.

In addition, publication bias is a concern in this meta-analysis of risk factors for NPM. In the current study, funnel plots and Egger’s test, two commonly used methods, were used to assess publication bias. The results of indicators such as nipple concavity, lactation mastitis, obesity, divorce or singleness, emotional problems, contraception and smoking were not statistically significant, suggesting that the possibility of publication bias in the current sample was relatively small. However, it must be clearly recognized that the absence of significant results in the Egger’s test does not absolutely exclude the existence of publication bias. After all, in reality, many factors may make it difficult for studies with negative results to be published, such as academic journals preferring to accept studies with positive conclusions, and the influence of researchers’ own submission strategies. Given these potential factors, the actual publication bias may not be fully detected.

Although the current research on the risk factors of NPM has revealed some valuable findings, there are still many limitations to be addressed. To further improve the understanding of the disease, future research should focus on the following key directions. In view of the fact that most of the existing studies are carried out in China, regional bias is prominent, and studies in other regions are needed. Samples from different countries and regions should be actively included, covering participants with diverse races, genetic backgrounds and living environments. Through multi-center collaborative research across regions, collecting comprehensive data can more accurately assess risk factors of NPM in different populations, reduce the bias caused by regional differences, and draw more universal and representative conclusions. All the included studies were retrospective case-control studies with no prospective studies. High-quality prospective cohort studies should be vigorously promoted in the future. Monitoring healthy people, regular detection and follow-up of relevant indicators, and recording the occurrence and development of the disease can more accurately determine the causal relationship and provide a strong basis for the formulation of prevention strategies. For risk factors with insufficient evidence, such as diabetes, heart disease and hypertension, more relevant research is needed to explore these factors in depth, expand the sample size, adopt advanced detection techniques and analysis methods, and investigate the potential association mechanism between these factors and NPM. Meanwhile, the combination of basic research and clinical practice should be strengthened, and the results of laboratory research should be transformed into clinically applicable diagnostic and therapeutic methods.

## Conclusion

NPM is a chronic breast disease characterized by recurrent episodes and long-lasting non-healing symptoms, severely impacting the physical and mental health of women. NPM prevention holds significant importance. This study has identified crater nipple, lactation mastitis, breast trauma, breastfeeding duration < 6 months, hyperprolactinemia, cardiopathy, hypertension, diabetes, obesity, contraception, smoking, emotional problems, and psychotropic drug use as risk factors for NPM. There is no clear association of NPM with galactostasis, allergic history, alcohol consumption, miscarriage, and divorce or being single. For individuals with multiple risk factors, regular breast follow-up is recommended. When breast symptoms occur, early diagnosis and treatment should be implemented based on the associated risk factors to avoid delay in managing the condition.

## Data Availability

The data used to support the findings of this study are included within the article.

## Abbreviations

NPM: Non-puerperal mastitis
CNKI: China National Knowledge Internet
PDM: Periductal mastitis
MDE: Mammary duct ectasia
IGM: Idiopathic granulomatous mastitis
NOS: Newcastle–Ottawa Scale
OR: Odds ratio
CI: Confidence interval
GLM: Granulomatous mastitis
